# Tumor Necrosis Factor-α Promotes Cholestasis-Induced Liver Fibrosis in the Mouse through Tissue Inhibitor of Metalloproteinase-1 Production in Hepatic Stellate Cells

**DOI:** 10.1371/journal.pone.0065251

**Published:** 2013-06-03

**Authors:** Yosuke Osawa, Masato Hoshi, Ichiro Yasuda, Toshiji Saibara, Hisataka Moriwaki, Osamu Kozawa

**Affiliations:** 1 Department of Pharmacology, Gifu University Graduate School of Medicine, Gifu, Gifu, Japan; 2 Department of Gastroenterology, Gifu University Graduate School of Medicine, Gifu, Gifu, Japan; 3 Faculty of Health Science, Suzuka University of Medical Science, Suzuka, Mie, Japan; 4 Department of Gastroenterology and Hepatology, Kochi University School of Medicine, Nankoku, Kochi, Japan; University of Birmingham, United Kingdom

## Abstract

Tumor necrosis factor (TNF)-α, which is a mediator of hepatotoxicity, has been implicated in liver fibrosis. However, the roles of TNF-α on hepatic stellate cell (HSC) activation and liver fibrosis are complicated and remain controversial. To explore this issue, the role of TNF-α in cholestasis-induced liver fibrosis was examined by comparing between TNF-α^−/−^ mice and TNF-α^+/+^ mice after bile duct ligation (BDL). Serum TNF-α levels in mice were increased by common BDL combined with cystic duct ligation (CBDL+CDL). TNF-α deficiency reduced liver fibrosis without affecting liver injury, inflammatory cell infiltration, and liver regeneration after CBDL+CDL. Increased expression levels of collagen α1(I) mRNA, transforming growth factor (TGF)-β mRNA, and α-smooth muscle actin (αSMA) protein by CBDL+CDL in the livers of TNF-α^−/−^ mice were comparable to those in TNF-α^+/+^ mice. Exogenous administration of TNF-α decreased collagen α1(I) mRNA expression in isolated rat HSCs. These results suggest that the reduced fibrosis in TNF-α^−/−^ mice is regulated in post-transcriptional level. Tissue inhibitor of metalloproteinase (TIMP)-1 plays a crucial role in the pathogenesis of liver fibrosis. TIMP-1 expression in HSCs in the liver was increased by CBDL+CDL, and the induction was lower in TNF-α^−/−^ mice than in TNF-α^+/+^ mice. Fibrosis in the lobe of TIMP-1^−/−^ mice with partial BDL was also reduced. These findings indicate that TNF-α produced by cholestasis can promote liver fibrosis via TIMP-1 production from HSCs. Thus, targeting TNF-α and TIMP-1 may become a new therapeutic strategy for treating liver fibrosis in cholestatic liver injury.

## Introduction

Chronic liver injury is characterized by hepatocyte cell death, hepatic inflammation, and activation of hepatic stellate cell (HSC), a major fibrogenic cell type in the liver [1]. Advanced liver fibrosis disrupts the normal architecture of the liver, causing hepatocellular dysfunction and portal hypertension. Cholestasis is associated with many liver diseases, and bile duct ligation (BDL) has been used in an animal model of chronic liver injury because it duplicates the hepatocyte damage, HSC activation, and liver fibrosis observed in human liver diseases. In the BDL model, accumulation of bile acids by biliary obstruction is thought to contribute to the liver damage [2], [3]. Bile acids are amphipathic molecules synthesized by hepatocytes and have detergent action required for lipid absorption. Exposure of hepatocytes to elevated concentrations of bile acid results in cell death [3], [4], and bile acid-associated death receptor-mediated cell death is one of the common mechanism for cholestatic hepatocyte injury [5]. Tumor necrosis factor (TNF)-α, which is the mediator of hepatotoxicity in many liver diseases [6], is elevated by common BDL (CBDL) [7], and the liver injury and fibrosis induced by CBDL are reduced in TNF-α^−/−^ mice [8] and TNF receptor (TNFR)1^−/−^ mice [9]. In addition, liver fibrosis induced by carbon tetrachloride (CCl_4_) is also reduced in TNFR1^−/−^ mice [10]. Thus, TNF-α has been thought to be crucial for liver injury and subsequent liver fibrosis. However, TNF-α alone does not induce hepatocyte cell death, and the sensitization of hepatocytes by D-galactosamine (GalN) is required for TNF-α-induced liver injury in mice [11], [12]. In contrast to its negative regulatory effects, TNF-α has a protective role in liver injury [13] and is required for liver regeneration [14]. Furthermore, TNF-α inhibits collagen α1(I) mRNA expression in HSCs [15], [16], [17]. Thus, the roles of TNF-α on HSCs activation and liver fibrosis are complicated and remain controversial.

Progression of liver fibrosis is associated with the inhibition of matrix degradation [18]. Matrix degradation is induced by the matrix metalloproteinase (MMP) family of enzymes; MMP-2, -3, and -9 are associated with the liver. Tissue inhibitor of metalloproteinase (TIMP)-1, the most important endogenous inhibitor of most MMPs, plays a crucial role in the pathogenesis of liver fibrosis, and its expression in HSCs is enhanced by TNF-α [9], [19], [20]. In human liver fibrosis, TIMP-1 expression is increased compared to that in the normal liver [18]. Overexpression of MMP-9 in the mouse liver, using an adenovirus vector, reduces liver fibrosis after CCl_4_ treatment [21]. Moreover, antagonization of TIMP-1 by a catalytically inactive mutant MMP-9 [21] or by TIMP-1 neutralizing antibody [22] decreases liver fibrosis. Inversely, transgenic mice overexpressing TIMP-1 in the liver show increased liver fibrosis after CCl_4_ treatment, whereas TIMP-1 overexpression alone does not result in liver fibrosis [23]. In addition to its role in inhibiting matrix degradation, TIMP-1 promotes survival and proliferation of liver cells. TIMP-1^−/−^ mice demonstrate impaired liver injury and hepatocyte proliferation after hepatic ischemia and reperfusion [24] and demonstrate exacerbated liver injury and fibrosis induced by CCl_4_ [25]. Thus, the effects of TIMP-1 on liver injury and fibrosis depend on pathophysiological condition, and its role on fibrosis after cholestatic liver injury remains unclear. To attempt to clarify the precise roles, this study investigated the involvement of TNF-α and TIMP-1 in the progression of fibrosis after cholestatic liver injury.

## Materials and Methods

### Ethics Statement

The experiments were conducted in accordance with the institutional guidelines and the protocol was approved by the Animal Research Committee of Gifu University (Permit Numbers: 23-3 and 23-38). All surgery was performed under anesthesia, and all efforts were made to minimize suffering.

### Animals

Wister male rats and male wild-type mice (C57Bl/6J), TNF-α-deficient mice (TNF-α^−/−^), and TIMP-1-deficient mice (TIMP-1^−/−^) were used for this study. TNF-α^−/−^ mice (#5540, C57Bl/6 background) and TIMP-1^−/−^ mice (#6243, C57Bl/6 background) were obtained from Jackson Laboratory (Bar Harbor, ME, USA), and wild-type C57Bl/6J mice and Wister male rats were from Japan SLC (Shizuoka, Japan).

### Bile Duct Ligation (BDL)

Eight-10 week-old male mice were used for studies. To perform common BDL and cystic duct ligation (CBDL+CDL), the peritoneal cavity was opened under anesthesia and the common bile duct was double ligated below the bifurcation, single ligated above the pancreas, and cut between the ligatures. In addition, the cystic duct was single ligated. The left hepatic duct was single ligated for partial BDL (PBDL) as previously reported [26], [27]. As necessary, GalN (Nacalai Tesque, Kyoto, Japan) (20 mg/mouse) was intraperitoneally administrated at 30 min before the surgery. On days 1, 3, 7, 21 after the surgery, mice were humanely killed.

### Measurement of Serum TNF-α

Mouse serum TNF-α level was measured by ELISA (Thermo Scientific, Rockford, IL, USA).

### Histological Analysis

The liver was fixed with 10% formalin, sectioned, and stained with H&E. Collagen deposition was stained with Sirius red (saturated picric acid containing 0.1% DirectRed 80 and 0.1% FastGreen FCF). The Sirius red positive area was quantitated using the ImageJ software (U.S. National Institutes of Health; http://rsb.info.nih.gov/ij/) and shown as a percentage of the total section area. Apoptosis was assessed by the terminal deoxynucleotidyl transferase-dUTP nick end labeling (TUNEL) assay (Promega, Madison, WI, USA, #G7132). The number of TUNEL positive nuclei was determined in 10 randomly selected fields. F4/80, CD3, Ki67, TIMP-1, and desmin were stained with anti-F4/80 (Santa Cruz Biotechnology, Santa Cruz, CA, USA, sc-52664), CD3 (Abcam, Cambridge, MA, USA, ab16669), Ki67 (Thermo scientific, RM-9106), TIMP-1 (R&D Systems, Minneapolis, MN, USA), desmin (Lab Vision, Fremont, CA, USA) antibodies using the Vectastain Elite ABC Kit (Vector Laboratories, Burlingame, CA, USA). Diaminobenzidine tetrahydrochloride was used as peroxidase substrate and sections were counterstained with hematoxylin. The immunostained-positive area of F4/80 was determined using ImageJ software and shown as a percentage of the total section area. The number of CD3 or Ki67-expressing cells was determined in 10 randomly selected fields. In some experiments, fluorescent-dye labeled secondary antibodies (Alexa Fluor 488 anti-rabbit for desmin and Alexa Fluor 594 anti-goat for TIMP-1) (Invitrogen, Carlsbad, CA, USA) were used for detection of primary antibodies as previously reported [28].

### Hydroxyproline Measurement

Hydroxyproline was measured for assessment of collagen content. The extracted liver protein was hydrolyzed in 6 M HCl (100°C, 24 h). The samples were neutralized with LiOH, and hydroxyproline was measured using a high-performance liquid chromatographic analyzer (Jasco, Hitachi, and Shimazu, Japan).

### Isolation of Rat Primary HSC

Rat primary HSCs were isolated as previously described [29]. The liver was perfused via the portal vein with collagenase (Wako, Osaka, Japan) and pronase E (EMD Chemicals, Gibbstown, NJ, USA). After digestion, the cell suspension was filtered through nylon mesh and purified via 8.2% Nycodenz (Axis-Shield, Oslo, Norway) gradient centrifugation. The isolated HSCs were cultured in uncoated plastic dishes with DMEM (Sigma-Aldrich, St. Louis, MO, USA) supplemented with 10% fetal bovine serum and antibiotic solution at 37°C in 5% CO_2_. After plating for 4 h, the medium was changed to DMEM with 10% fetal bovine serum and antibiotics containing TNF-α (30 ng/ml, R&D Systems) for 72 h. The purity of HSCs was always 95% as determined by their typical starlike shape and abundant lipid droplets with vitamin A autofluorescence.

### Western Blot

Electrophoresis of protein extracts and blotting were performed with anti-cyclin E (Santa Cruz Biotechnology, sc-481), glyceraldehyde-3-phosphate dehydrogenase (GAPDH) (Cell Signaling Technology, Danvers, MA, USA, #2118), α-smooth muscle actin (αSMA) (Sigma-Aldrich, A2547), and TIMP-1 antibodies. Then, the membrane was incubated with the horseradish peroxidase-coupled secondary antibodies (Santa Cruz Biotechnology). Detection was performed with an ECL system (Amersham Biosciences, Buckinghamshire, UK), and the protein bands were quantified by densitometry using the ImageJ software.

### Quantitative Real Time RT-PCR

RNA was extracted from liver tissue and cultured cells using the RNeasy and DNase Kits (Qiagen, Valencia, CA, USA) and was reverse-transcribed using the High-Capacity cDNA Reverse Transcription Kit (Applied Biosystems, Foster City, CA, USA). Quantitative real-time RT-PCR was performed using the SYBR Premix Ex Taq (Takara, Shiga, JAPAN) for mouse and rat TIMP-1 (forward; TGGGGAACCCATGAATTTAG, reverse; TCTGGCATCCTCTTGTTGC), rat collagen type I α1 (forward; TAGGCCATTGTGTATGCAGC, reverse; ACATGTTCAGCTTTGTGGACC), mouse αSMA (forward; GTTCAGTGGTGCCTCTGTCA, reverse; ACTGGGACGACATGGAAAAG), rat αSMA (forward; GTTCAGCGGCGCCTCCGTTA, reverse; ACTGGGACGACATGGAAAAG), rat and mouse desmin (forward; CTCGGAAGTTGAGAGCAGAGA, reverse; GTGAAGATGGCCTTGGATGT), mouse vimentin (forward; ACCGCTTTGCCAACTACAT, reverse; TTGTCCCGCTCCACCTC), and rat chemokine (C-C motif) ligand 5 (CCL5) (forward; CCACTTCTTCTCTGGGTTGG, reverse; GTGCCCACGTGAAGGAGTAT), and probe-primers sets (Applied Biosystems) for mouse procollagen type I α1 (Mm00801666g1), mouse transforming growth factor (TGF)-β1 (Mm00441724m1) and 18S ribosomal RNA (Hs99999901s1) with the LightCycler 480 (Roche Applied Science, Mannheim, Germany). The changes were normalized based on 18S rRNA values.

### Gelatin Zymography

Gelatin zymography was performed with extracted proteins from the liver (50 µg) as described previously [30]. Frozen livers were homogenized in lysis buffer (20 mmol/L HEPES [pH 7.5], 150 mmol/L NaCl, 10 mmol/L CHAPS) and the homogenates were centrifuged at 20,000*g* for 20 min at 4°C. Proteins from supernatant were separated in 7.5% polyacrylamide gel containing 1 mg/mL of gelatin. The gels were equilibrated in developing buffer (50 mmol/L Tris [pH 7.4], 200 mmol/L NaCl, 10 mmol/L CaCl2, 0.02% sodium azaid). The gel was stained with 0.5% Coomassie Blue R-250, followed by destaining. Gelatinolytic activity was detected as clear bands on a dark blue background.

### Statistical Analysis

Data are expressed as the mean ± SD of data collected from at least 5 independent experiments. Data between groups were analyzed by the 2-tailed Student’s *t*-test. A *P* value of less than 0.05 was an indication of statistical significance.

## Results

### Deficiency of TNF-α Reduces CBDL+CDL-induced Liver Fibrosis

**Figure 1:**
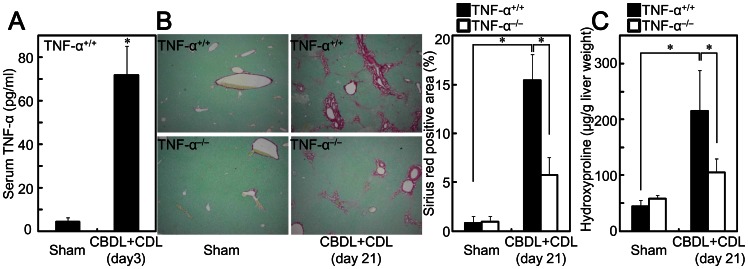
TNF-α deficiency reduced liver fibrosis after CBDL+CDL. TNF-α^+/+^ and TNF-α^−/−^ mice received CBDL+CDL. The animals were sacrificed on 3 (A) or 21 (B, C) days after the surgery. (A) Serum TNF-α levels were measured by ELISA. (B, C) Collagen deposition was assessed by Sirius red staining (B, original magnification: 40×, graph in right panel) and measurement of hydroxyproline content (C). Data are mean ± SD from at least 5 independent experiments. *, P<0.05 using a 2-tailed Student’s t-test.

Serum TNF-α level was increased by CBDL+CDL in wild-type mice (Figure 1A), as previously reported [7]. To explore the roles of TNF-α on liver fibrosis, CBDL+CDL was performed on TNF-α^−/−^ mice. Fibrosis was induced in CBDL+CDL mice, as demonstrated by Sirius red staining and hydroxyproline content (Figure 1B, C). CBDL +CDL livers of TNF-α^−/−^ mice showed reduced fibrosis, compared to those of TNF-α^+/+^ mice (Figure 1B, C), suggesting that TNF-α contributes to liver fibrosis.

**Figure 2:**
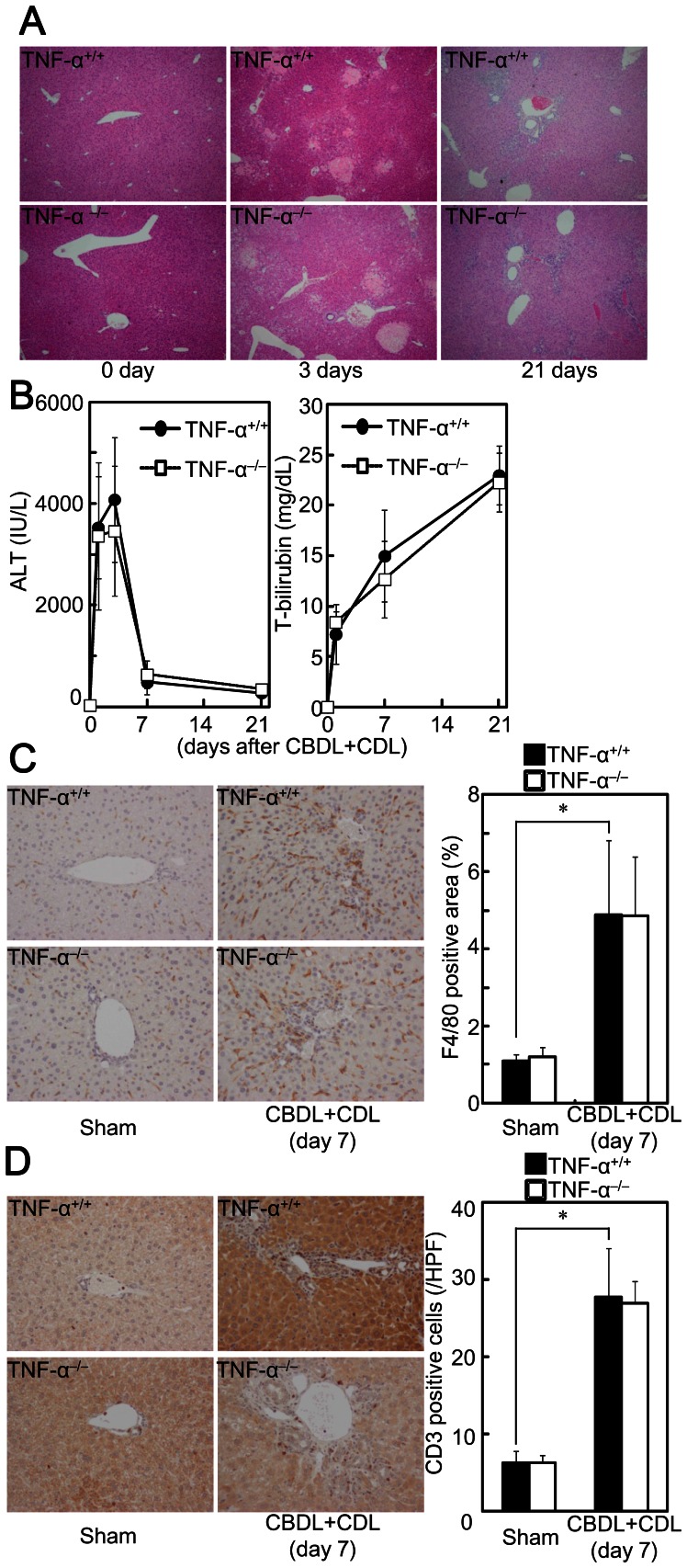
TNF-α deficiency did not affect CBDL+CDL-induced liver injury. TNF-α^+/+^ and TNF-α^−/−^ mice received CBDL+CDL. The animals were sacrificed at the indicated times. (A) The injured lesion in the livers was assessed by H&E staining (original magnification: 40×). (B) Serum ALT and total bilirubin levels were compared at the indicated times. (C) Expression of F4/80 in the livers was examined by immunohistochemistry (original magnification: 200×). F4/80 positive area was compared (right panel). (D) CD3^+^ cells in the livers were examined by immunohistochemistry (original magnification: 200×). Number of CD3^+^ cells was compared. Data are mean ± SD from at least 5 independent experiments. *, P<0.05 vs. sham using a 2-tailed Student’s t-test.

**Figure 3:**
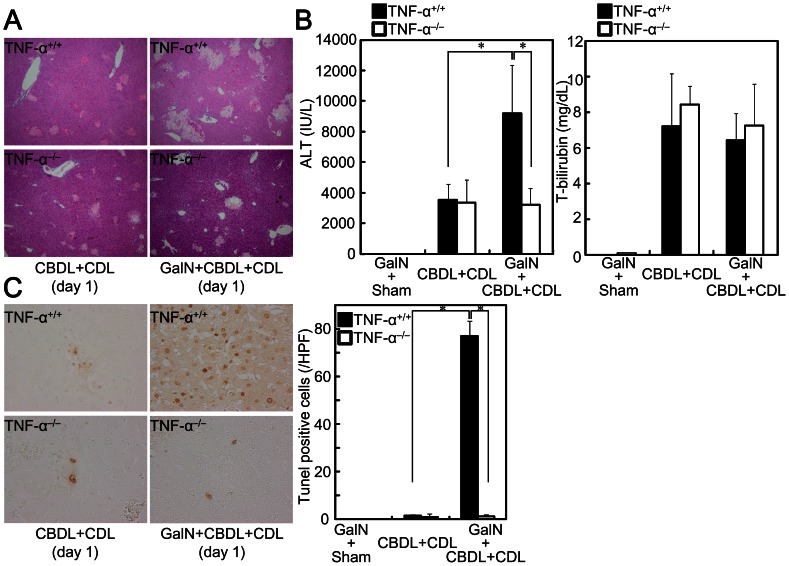
TNF-α-mediated increase of liver injury and hepatocyte apoptosis after CBDL+CDL was induced only in GalN sensitized mice. TNF-α^+/+^ and TNF-α^−/−^ mice were treated with or without GalN (20 mg) and subjected to CBDL+CDL. The animals were sacrificed 24 h after the surgery. (A) The injured lesions in the livers were assessed by H&E staining (original magnification: 40×). (B) Serum ALT and total bilirubin levels were compared. (C) Apoptotic nuclei were identified by TUNEL staining (original magnification: 400×). Numbers of TUNEL-positive cells were compared (right panel). Data are mean ± SD from at least 5 independent experiments. *, P<0.05 using a 2-tailed Student’s t-test.

**Figure 4:**
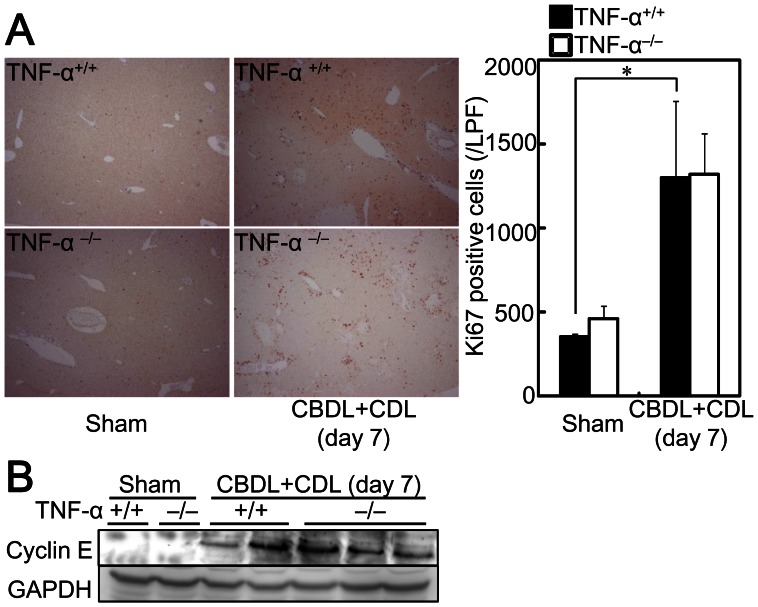
TNF-α deficiency did not affect hepatocyte regeneration after CBDL+CDL. TNF-α^+/+^ and TNF-α^−/−^ mice received CBDL+CDL. The animals were sacrificed 7 days after the surgery. (A) Expression of Ki67 in the livers was examined by immunohistochemistry (original magnification: 40×). Number of Ki67^+^ cells was compared (right panel). Data are mean ± SD from at least 5 independent experiments. *, P<0.05 using a 2-tailed Student’s t-test. (B) The protein extracts from the livers were subjected to SDS-PAGE and immunoblotting was performed with anti-cyclin E and -GAPDH antibodies. The results shown are representative of at least 3 independent experiments.

Liver fibrosis is induced by liver cell damage and inflammatory cell infiltration with impaired hepatocyte regeneration. However, TNF-α^−/−^ mice showed liver injury (Figure 2A), increased serum alanine aminotransferase (ALT) and total bilirubin (Figure 2B), infiltrated F4/80^+^ macrophages (Figure 2C), and number of infiltrated CD3^+^ lymphocytes (Figure 2D) after CBDL +CDL to a similar degree as was observed for TNF-α^+/+^ mice, suggesting that TNF-α is not related to liver injury and inflammatory cell infiltration in CBDL+CDL mice. To confirm the irrelevance of TNF-α to liver injury, the mice were pretreated with GalN, which increases sensitivity to TNF-α-induced liver damage and hepatocyte apoptosis [31], and subsequently received CBDL+CDL. GalN treatment alone did not induce liver injury or fibrosis (data not shown). Liver injury by CBDL+CDL was exacerbated in the GalN-pretreated mice (Figure 3A). Serum ALT levels were also higher in the GalN-pretreated mice than in the non-treated mice, although total bilirubin was comparable (Figure 3B), suggesting that hepatocyte cell death was exacerbated in the GalN-pretreated mice without increased cholestasis. Moreover, TUNEL-positive hepatocytes were increased in GalN-treated mice that received CBDL+CDL (Figure 3C). These effects of GalN were blunted in TNF-α^−/−^ mice (Figure 3). TNF-α treatment alone does not induce hepatocyte cell death, and the sensitization of hepatocytes by GalN is required for TNF-α-induced liver injury in mice [11], [12]. These previous findings, in conjunction with our results, suggest that the liver damage increments caused by GalN combination is induced by the TNF-α produced by CBDL+CDL; TNF-α causes liver damage only when the hepatocytes are sensitized by GalN in CBDL+CDL mice, and TNF-α does not contribute to liver damage in CBDL+CDL without GalN. Thus, this liver damage may be primarily induced by accumulated cytotoxic bile acids. Furthermore, CBDL+CDL-mediated induction of Ki67^+^ cells (Figure 4A) and elevation of cyclin E (Figure 4B), which are makers for liver regeneration, in the livers of TNF-α^−/−^ mice was comparable to that in TNF-α^+/+^ mice, suggesting that liver regeneration after CBDL+CDL was not mediated by the produced TNF-α. Thus, the reduced fibrosis in TNF-α^−/−^ mice after CBDL+CDL was not related to the reduction of liver damage or the enhancement of liver regeneration.

### TNF-α Decreases Collagen mRNA Expression in Isolated rat HSCs

**Figure 5:**
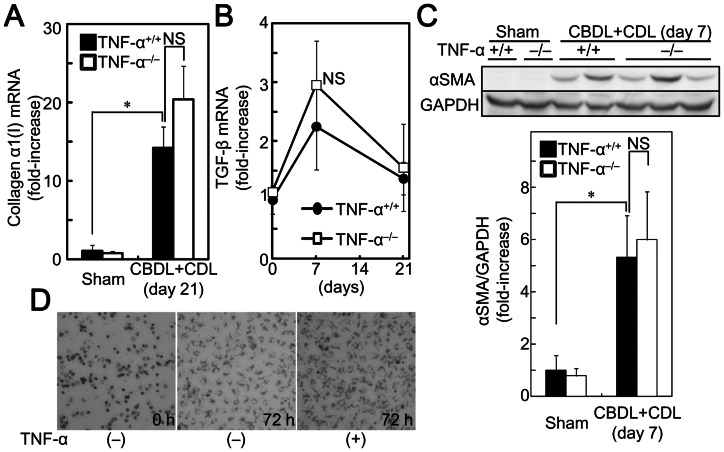
TNF-α deficiency did not affect collagen α1(I) mRNA expression and HSC activation in the livers of mice after CBDL+CDL.

Because fibrosis was decreased in TNF-α^−/−^ mice, it is possible that TNF-α activates HSCs and increases their collagen production. Indeed, it is reported that TNF-α activates primary cultured HSCs by activation of p38 mitogen-activated protein kinase (MAPK) and c-jun N-terminal kinase (JNK) [32]. To compare the activity status of HSCs between TNF-α^+/+^ and TNF-α^−/−^ mice, we examined expression levels of collagen α1(I) mRNA, TGF-β mRNA, which is an activator of HSCs, desmin and vimentin mRNA, which are classical features of HSCs, and αSMA mRNA and protein, which is a maker for stellate cell activation. The increased CBDL+CDL-mediated expression levels of collagen α1(I) mRNA (Figure 5A), TGF-β mRNA (Figure 5B), αSMA mRNA, desmin mRNA, vimentin mRNA (Table 1), and αSMA protein (Figure 5C) in the livers of TNF-α^−/−^ mice were comparable to those of TNF-α^+/+^ mice, suggesting that TNF-α does not contribute to activation of HSCs and production of collagen in CBDL+CDL mice. To examine the direct effects of TNF-α on collagen expression in HSCs, primary HSCs were isolated from rats and were cultured on plastic dishes, on which the cells were automatically activated and proliferated (Figure 5D). According to the autoactivation, expression levels of collagen α1(I), αSMA, and desmin mRNA in HSCs were increased by culture on plastic dishes for 72 h (Table 2). Exogenous administration of TNF-α decreased the induction of collagen α1(I) mRNA as previously reported [15], [16], [17], although αSMA was not reduced by TNF-α. Moreover, TNF-α increased desmin and CCL5 mRNA in HSCs (Table 2) which is a mediator of HSC proliferation [33]. Thus, TNF-α decreases collagen α1(I) production without inhibition of activation and proliferation in primary cultured HSCs. These results further suggest that the reduced fibrosis in TNF-α^−/−^ mice is not due to reduction of collagen synthesis in HSCs.

**Table 1:** Changes in the mRNA profiles of the liver after CBDL+CDL in TNF-α^−/−^ mice TNF-α^+/+^ and TNF-α^−/−^ mice received CBDL+CDL.

	sham		CBDL+CDL	
	TNF-α^+/+^	TNF-α^−/−^	TNF-α^+/+^	TNF-α^−/−^
αSMA	1.00±0.64	1.36±0.25	5.43±0.98*	6.32±1.22
desmin	1.00±0.36	1.19±0.17	1.73±0.28*	1.76±0.69
vimentin	1.00±0.18	1.22±0.23	2.77±0.82*	3.66±0.77

The animals were sacrificed 7 days after the surgery. mRNA levels of αSMA, desmin, and vimentin in the livers were determined by quantitative real time RT-PCR. Results are presented as means ± SD of data collected from at least 5 independent experiments.

* P<0.05 versus sham-operated TNF-α^+/+^ mice using a 2-tailed Student’s t-test.

**Table 2:** Effect of TNF-α on mRNA profiles of primary isolated rat HSCs.

Incubation time	0 h	72 h	72 h
TNF-α	(–)	(–)	(+)
collagen α1(I)	1.00±0.12	7.95±0.85	3.52±0.45*
αSMA	1.00±0.16	7.44±0.69	6.71±0.78
desmin	1.00±0.17	2.20±0.11	4.32±0.44*
CCL5	1.00±0.23	0.15±0.02	1.63±0.25*
TIMP-1	1.00±0.07	2.25±0.22	3.76±0.33*

Primary rat HSCs were incubated on plastic dishes for 72 h with or without TNF-α (30 ng/mL). mRNA levels of collagen α1(I), αSMA, desmin, CCL5, and TIMP-1 were determined by quantitative real time RT-PCR (E). Data are mean ± SD from at least 6 independent experiments.

* P<0.05 versus 72 h cultured HSCs without TNF-α using a 2-tailed Student’s t-test.

### TIMP-1 is Induced in BDL Livers in a TNF-α-dependent Manner

**Figure 6:**
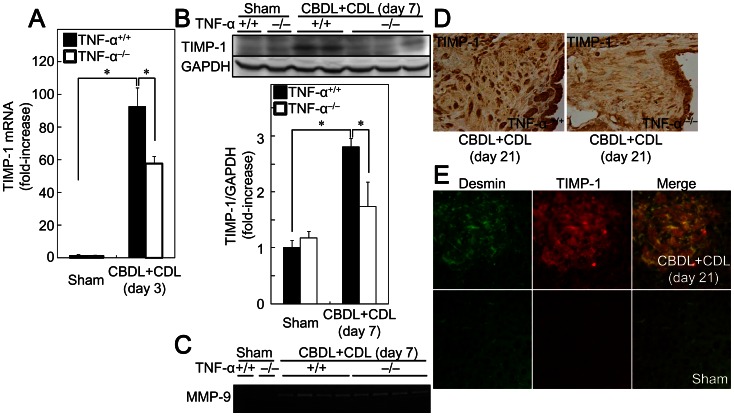
CBDL+CDL increased TIMP-1 in a TNF-α dependent manner. TNF-α^+/+^ and TNF-α^−/−^ mice received CBDL+CDL. The animals were sacrificed at the indicated times. (A) mRNA levels of TIMP-1 in the livers were determined by quantitative real time RT-PCR. (B) The protein extracts from the livers were analyzed by SDS-PAGE, and immunoblotting was performed with anti-TIMP-1 and -GAPDH antibodies. The results shown are representative of at least 5 independent experiments. Relative densitometric intensity of TIMP-1 was determined for each protein band and normalized to GAPDH (bottom panels). (C) Collagenase activities in the protein extracts were measured by gelatin zymography. (D) Expression of TIMP-1 in the livers was examined by immunohistochemistry (original magnification: 400×). (E) Expression of desmin (green) and TIMP-1 (red) around the interstitial space around dilated bile ducts was examined by immunofluorescent staining. The results shown are representative of at least 3 independent experiments. Data are mean ± SD from at least 5 independent experiments. *, P<0.05 using a 2-tailed Student’s t-test.

After finding that collagen mRNA was not decreased in TNF-α^−/−^ mice, we hypothesized that the reduced fibrosis in the mice was due to post-transcriptional effects. To elucidate the mechanisms by which TNF-α contributes to BDL-mediated liver fibrosis, we focused on TIMP-1, which is an endogenous inhibitor of MMPs. Consistent with a report indicating that a TNF-α inhibitor can prevent the increase in rat hepatic TIMP-1 by CCl_4_ treatment [34], we found that CBDL+CDL increased mRNA (Figure 6A) and protein (Figure 6B) expression of TIMP-1 in the liver, and the induction was lower in TNF-α^−/−^ mice than in TNF-α^+/+^ mice. In contrast, gelatin zymography showed that MMP-9 (Figure 6C) and MMP-2 (data not shown) were equally activated by CBDL+CDL in both TNF-α^+/+^ and TNF-α^−/−^ mice. TIMP-1 positive cells were induced in the increased interstitial space around the dilated bile ducts (Figure 6D) and most of the desmin^+^ cells (a marker of HSCs) showed double staining for TIMP-1 (Figure 6E), suggesting that TIMP-1 is expressed by HSCs. In addition, TIMP-1 mRNA expression in primary cultured rat HSCs was increased by TNF-α administration (Table 2), indicating that TNF-α stimulates TIMP-1 production, as previously reported [9], [19], [20]. These results suggest that the TNF-α produced by BDL induces TIMP-1 in HSCs.

### Fibrosis is Reduced in TIMP-1^−/−^ Mice

**Figure 7:**
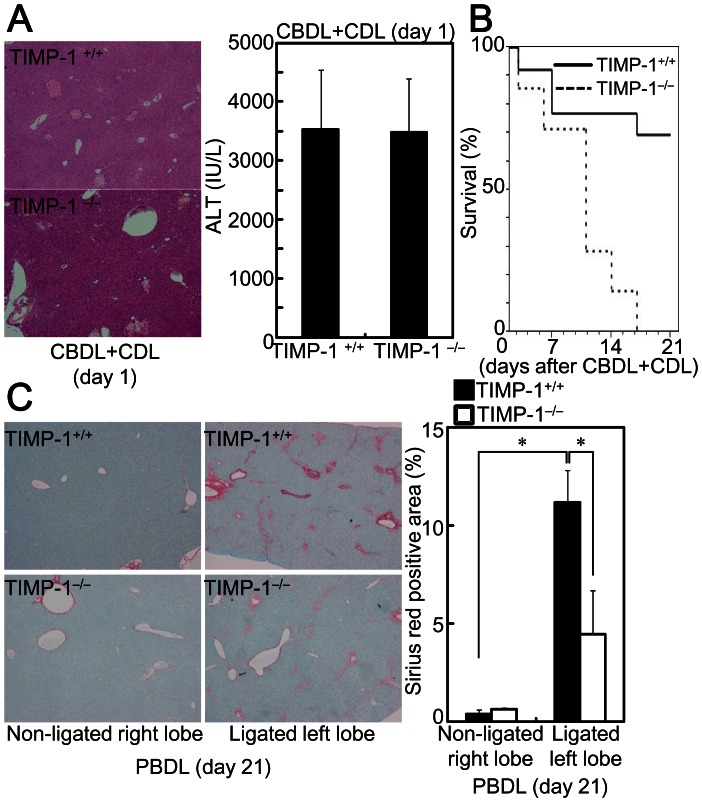
TIMP-1 deficiency reduced liver fibrosis after BDL. TIMP-1^+/+^ and TIMP-1^−/−^ mice received CBDL+CDL (A, B) or PBDL (C). The animals were sacrificed at the indicated times. (A) The injured lesions in the livers were assessed by H&E staining (original magnification: 40×, left panel). Serum ALT levels were compared (right panel). (B) Survival curves for animals with CBDL+CDL. (C) Collagen deposition in the ligated left robes was assessed by Sirius red staining (original magnification: 40×). Sirius red positive area was compared (right panel). Data are mean ± SD from at least 5 independent experiments. *, P<0.05 using a 2-tailed Student’s t-test.

Induction of TIMP-1, but not MMPs, was reduced in TNF-α^−/−^ mice. This led to the hypothesis that insufficient TIMP-1 expression leads to increased collagen removal by MMPs in TNF-α^−/−^ mice. To investigate the roles of TIMP-1 on fibrosis, we performed BDL on TIMP-1^−/−^ mice. TIMP-1^−/−^ mice showed a similar degree of increased liver injury and serum ALT (Figure 7A) as TIMP-1^+/+^ mice at 1 day after CBDL+CDL. In addition, the increased expression levels of αSMA, desmin, and vimentin mRNA in the livers of TIMP-1^−/−^ mice at 7 days after CBDL+CDL were comparable to those in TIMP-1^+/+^ mice (Table 3). These results suggest that TIMP-1 does not play a role in hepatocyte cell death induced by bile acid or in activation of HSCs in CBDL+CDL mice. Surprisingly, the mortality rate of TIMP-1^−/−^ mice that received CBDL+CDL was extremely high; 0% of mice remained by 21 days after the surgery (Figure 7B). Mice treated with our established model, PBDL, only showed liver injury and fibrosis in the bile duct ligated left lobe of the liver, which improved the survival rate to 100% in both the TIMP-1^+/+^ and TIMP-1^−/−^ mice. Therefore, we used PBDL for the TIMP-1^−/−^ mice. Sirius red staining showed a smaller positive area in the ligated left lobe of the TIMP-1^−/−^ mice (Figure 7C), suggesting that fibrosis is reduced by a TIMP-1 deficiency. Thus, TIMP-1 may have fibrogenic effects due to inhibition of collagen removal by MMPs.

**Table 3:** Changes in the mRNA profiles of the liver after CBDL+CDL in TIMP-1^−/−^ mice TIMP-1^+/+^ and TIMP-1^−/−^ mice received CBDL+CDL.

	sham		CBDL+CDL	
	TIMP-1^+/+^	TIMP-1^−/−^	TIMP-1^+/+^	TIMP-1^−/−^
αSMA	1.00±0.64	1.04±0.30	5.43±0.98*	4.01±1.72
desmin	1.00±0.36	1.39±0.18	1.73±0.28*	1.59±0.34
vimentin	1.00±0.18	1.19±0.29	2.77±0.82*	2.05±0.86

The animals were sacrificed 7 days after the surgery. mRNA levels of αSMA, desmin, and vimentin in the livers were determined by quantitative real time RT-PCR. Results are presented as means ± SD of data collected from at least 5 independent experiments.

* P<0.05 versus sham-operated TIMP-1^+/+^ mice using a 2-tailed Student’s t-test.

## Discussion

The present study investigated the contribution of TNF-α to the progression of fibrosis after cholestatic liver injury. The results, which indicate that TNF-α increases liver fibrosis through TIMP-1 production from HSCs, suggest novel therapeutic possibilities for treating liver fibrosis.

TNF-α has been thought to be crucial for liver injury and fibrosis by BDL because those are reduced in TNF-α^−/−^ mice [8] and TNFR1^−/−^ mice [9]. Gabele et al. reported that TNF-α^−/−^ mice display reduced levels of serum ALT, Sirius red positive area, collagen-I protein expression, αSMA positive cells, and TGF-β mRNA in the liver after BDL [8]. After BDL, hepatocytes are exposed to elevated concentrations of bile acid, and hydrophobic bile acids lead to hepatocyte cell death [3], [4] through various factors, such as reactive oxygen species (ROS) generation from mitochondria [35]. The initial hepatocyte cell death stimulates subsequent inflammatory responses, leading to further liver injury and fibrosis [36], [37]. TNF-α is induced by BDL after the initial hepatocellular damage caused by bile acids, and both of bile acids and the induced TNF-α are reported to induce hepatocellular damage. Among them, the TNF-α-induced liver injury is canceled in TNF-α^−/−^ mice after BDL. Because liver injury stimulates HSC activation, reduced liver injury may decrease fibrosis. However, it is still unclear whether the reduced liver fibrosis is due to a lack of fibrogenic effects of TNF-α or due to the reduced liver injury because previous reports indicate that both liver injury and fibrosis after CBDL are reduced in TNF-α^−/−^ mice [8] and TNFR1^−/−^ mice [9]. In contrast to those reports, our study found a reduction of liver fibrosis after CBDL+CDL in TNF-α^−/−^ mice without a reduction in liver injury. This can be attributed to this study’s use of CBDL+CDL, which is composed of CBDL and CDL, in contrast to the usual CBDL procedure, in which only the common bile duct is ligated, used in the previous studies. In CBDL+CDL mice, congested bile did not remain in the gall bladder, leading to the post-surgical exposure of hepatocytes to high concentrations of bile acid, which induced severe hepatocyte cell death. CBDL+CDL induced same degree of liver damage in TNF-α^−/−^ compared with TNF-α^+/+^ suggesting that the liver injury induced by bile acid may be severe enough to conceal TNF-α-mediated liver injury. In actuality, pretreatment with GalN exacerbated the CBDL+CDL-induced liver injury that was blunted in TNF-α^−/−^ mice, indicating that TNF-α has a minor role in liver injury in CBDL+CDL mice. TNF-α^−/−^ mice showed reduced fibrosis after CBDL+CDL, without reduction of liver injury and inflammatory cell infiltration or enhancement of liver regeneration, indicating a direct contribution by TNF-α to liver fibrosis in CBDL+CDL mice.

The profibrogenic effects of TNF-α in another fibrosis model, which uses CCl_4_, have been reported previously, indicating that TNFR1 deficiency inhibits liver fibrosis after CCl_4_ treatment, without any effects on liver injury [10], [38]. TGF-β, one of the main fibrogenic factors, stimulates HSCs to induce collagen I α1 transcription [39]. However, the induction of TGF-β and collagen mRNAs after CBDL+CDL was similar in TNF-α^−/−^ and TNF-α^+/+^ mice. Moreover, exogenous administration of TNF-α decreased the induction of collagen α1(I) mRNA in primary cultured rat HSCs. These results suggest that TNF-α induces liver fibrosis post-transcriptionally. MMPs and their inhibitor, TIMP, post-translationally modulate ECM remodeling, and the balance of their expression plays an important role in liver fibrosis [18]. Indeed, CBDL+CDL increased TIMP-1 expression, which can inhibit a broad range of MMPs and was attenuated by TNF-α deficiency. In addition, fibrosis in the ligated lobes of PBDL livers was reduced in TIMP-1^−/−^ mice compared to that in TIMP-1^+/+^ mice. Thus, insufficient TIMP-1 production is a possible explanation for the reduced fibrosis in TNF-α^−/−^ mice after CBDL+CDL. In contrast to our results, it has been reported that CCl_4_-induced liver fibrosis and liver injury are increased in TIMP-1^−/−^ mice [25]. In addition to its profibrogenic effects, TIMP-1 has a protective effect on hepatocytes [25]. Inhibition of MMPs blocks apoptosis of hepatocytes [40]. TIMP-1^−/−^ mice demonstrate impaired liver injury and hepatocyte proliferation after hepatic ischemia and reperfusion [24]. Thus, deletion of TIMP-1 could accelerate liver fibrosis by increasing liver injury. Indeed, TIMP-1^−/−^ mice that received CBDL+CDL showed an extremely high mortality rate, with an initial liver injury rate that was comparable to TIMP-1^+/+^ mice, suggesting that TIMP-1 has roles in the BDL liver, in addition to MMP inhibition. However, our results using PBDL suggest that TIMP-1 has a profibrogenic role in cholestatic liver injury. TIMP-1 can act directly through cell surface receptors, in addition to indirectly directing cell fate through modulation of protease activity [41]. Both stimulatory [24] and inhibitory [42] effects of TIMP-1 on liver regeneration have been reported. Thus, further studies are required to investigate the multifunctional effects of TIMP-1 on liver injury and fibrosis. In addition, the mechanism of TIMP-1 up-regulation induced by TNF-α in the BDL liver is still unclear. It has been reported that activation of HSCs is accompanied by induction of TIMP-1 promoter activity and mRNA expression, and that AP-1, Pea3, and TIMP-1 element 1 are reported to be involved in transcriptional activity of the TIMP-1 promoter [43], [44], [45]. Moreover, TIMP-1 expression is also regulated by changes in mRNA stability [46]. Further studies are also needed to resolve this uncertainty.

In conclusion, we observed that TNF-α produced by cholestasis promoted liver fibrosis via TIMP-1 production from HSCs. Thus, targeting TNF-α and TIMP-1 may become a new therapeutic strategy for treating liver fibrosis and cholestatic liver injury.
